# Analgesic Effect of Intraoperative Intravenous S(+)-Ketamine During Total Knee Arthroplasty Surgery: Study Protocol for a Randomized Controlled Clinical Trial

**DOI:** 10.2196/53063

**Published:** 2023-12-08

**Authors:** Shiyuan Deng, Lina Chen, Xiao Song, Liang Guo, Fei Zhao, Jing Liu, Ling Dong

**Affiliations:** 1 Department of Anesthesiology The First Affiliated Hospital of Shandong First Medical University Jinan China

**Keywords:** analgesic, S(+)-ketamine, total knee arthroplasty surgery, older patients, multimodal pain management

## Abstract

**Background:**

Total knee arthroplasty (TKA) is currently the main treatment for end-stage knee disease. The number of cases of TKA in China increased from 53,880 in 2011 to 374,833 in 2019, representing a 5.9-fold increase. Moderate to severe pain often occurs after TKA, which seriously affects postoperative rehabilitation, patient satisfaction, and overall outcome. Multimodal analgesia is considered the ideal solution. Adequate postoperative analgesia can not only reduce pain, opioid consumption, and, consequently, opioid-related adverse events, but can also reduce the length of hospital stay and costs and improve rehabilitation and patient satisfaction.

**Objective:**

Effective multimodal pain management in the early postoperative period is essential for anesthesiologists. Additional studies have demonstrated that a low-dose intravenous infusion of ketamine can be administered as an adjuvant drug to alleviate acute postoperative pain. Therefore, we aim to appraise the efficacy and safety of intraoperative intravenous injection of S(+)-ketamine to relieve acute pain after TKA in older patients.

**Methods:**

This is a protocol for a randomized, placebo-controlled trial. A total of 144 participants aged 65 years and older undergoing TKA will be randomly allocated into the S(+)-ketamine and placebo groups in a 1:1 ratio. S(+)-ketamine or the placebo will be intravenously administered at 0.3 mg/kg/h during the operation by anesthesiologists. Blinded evaluation by trained investigators will be completed at 2 hours, 24 hours, and 48 hours after surgery. The primary outcome measure will be the numeric rating scale score at rest and movement at 24 hours after surgery. The secondary outcomes will include the numeric rating scale scores at rest and movement at 2 hours and 48 hours after surgery, the number of patients who require additional analgesics during the first 48 hours after operation, the total consumption of opioids or nonsteroid anti-inflammatory drugs during the first 48 hours after operation, and adverse events at 2, 24, and 48 hours after operation.

**Results:**

The protocol was registered at Clinical Trials.gov on February 26, 2022. It was performed in accordance with the approved guidelines and regulations of the participating institutions. Recruitment began in April 2022. Data collection is expected to conclude in September 2024. Study completion is expected in December 2024.

**Conclusions:**

A randomized, controlled, clinical study was designed to observe the analgesic effect of intraoperative intravenous administration of a lower dose of S(+)-ketamine (0.3 mg/kg/h) in older patients after TKA surgery. The protocol will appraise the efficacy and safety of intraoperative intravenous injection of S(+)-ketamine to relieve acute pain after TKA in older patients who may benefit from the administration of S(+)-ketamine.

**Trial Registration:**

ClinicalTrials.gov NCT05289050; https://clinicaltrials.gov/ct2/show/NCT05289050

**International Registered Report Identifier (IRRID):**

DERR1-10.2196/53063

## Introduction

Total knee arthroplasty (TKA) is currently the main treatment for end-stage knee disease. The number of cases of TKA in China increased from 53,880 in 2011 to 374,833 in 2019, representing a 5.9-fold increase [1]. Moderate to severe pain often occurs after TKA [2], which seriously affects postoperative rehabilitation, patient satisfaction, and overall outcomes [3,4]. Multimodal analgesia is considered the ideal solution. Adequate postoperative analgesia can not only reduce pain, opioid consumption, and, consequently, opioid-related adverse events, but can also reduce the length of hospital stay and costs and improve rehabilitation and patient satisfaction [5].

Opioids are effective for acute postoperative pain, but they have numerous adverse effects (eg, respiratory depression and nausea), especially in older patients. In addition, the use of excessive opioids may pose a risk of opioid addiction [6]. Despite encouraging results and rapid recovery after the use of perioperative local infiltration analgesia and nerve block agents, pain management can be further optimized [7]. Therefore, effective multimodal pain management in the early postoperative period is essential for anesthesiologists.

Ketamine has been widely used in anesthesia to relieve postoperative pain. It mainly acts on the N-methyl-D-aspartate (NMDA) receptor to produce sedative, analgesic, and anesthetic effects [8,9]. S(+)-ketamine is an effective S-enantiomer of racemic ketamine with a greater affinity than ketamine (approximately 2- to 4-fold) for NMDA receptors, thereby producing stronger sedative and analgesic effects and fewer adverse events, and it has been used to prevent central sensitization [10-13]. Opioids, such as remifentanil, may lead to hyperalgesia and more analgesic requirements. A clinical study showed that IV ketamine improved the analgesic effect of fentanyl and lessened hyperalgesia [14].

Aggressive management of TKA pain has multiple benefits, such as shortening the length of hospital stay, preventing postoperative complications, and accelerating patient recovery [15]. Therefore, S(+)-ketamine requires further investigation, and the following questions must be addressed: What is the ideal dosage of S(+)-ketamine infusion for the prevention of postoperative acute pain? Can older patients experience a beneficial effect after surgery? To answer these questions, a randomized, controlled, clinical study was designed to observe the analgesic effect of intraoperative intravenous administration of a lower dose of S(+)-ketamine (0.3 mg/kg/h) in older patients after TKA surgery.

## Methods

### Objective and Trial Design

This clinical study was designed to observe the analgesic effect of a lower intravenous dose of S(+)-ketamine (0.3 mg/kg/h) in patients after TKA surgery.

This is a single-center, randomized, single-blind, controlled, clinical trial in older patients undergoing TKA under general anesthesia. Eligible patients will be randomized to receive S(+)-ketamine (n=72) or a placebo equivalent, 0.9% sodium chloride (n=72), in a ratio of 1:1. Patients will be randomized using a computer-generated list. The anesthetist nurse and the pharmacist will not be blinded and will not partake in the following research, but the follow-up evaluators will be blinded in the trial.

### Recruitment and Study Setting

**Figure 1:**
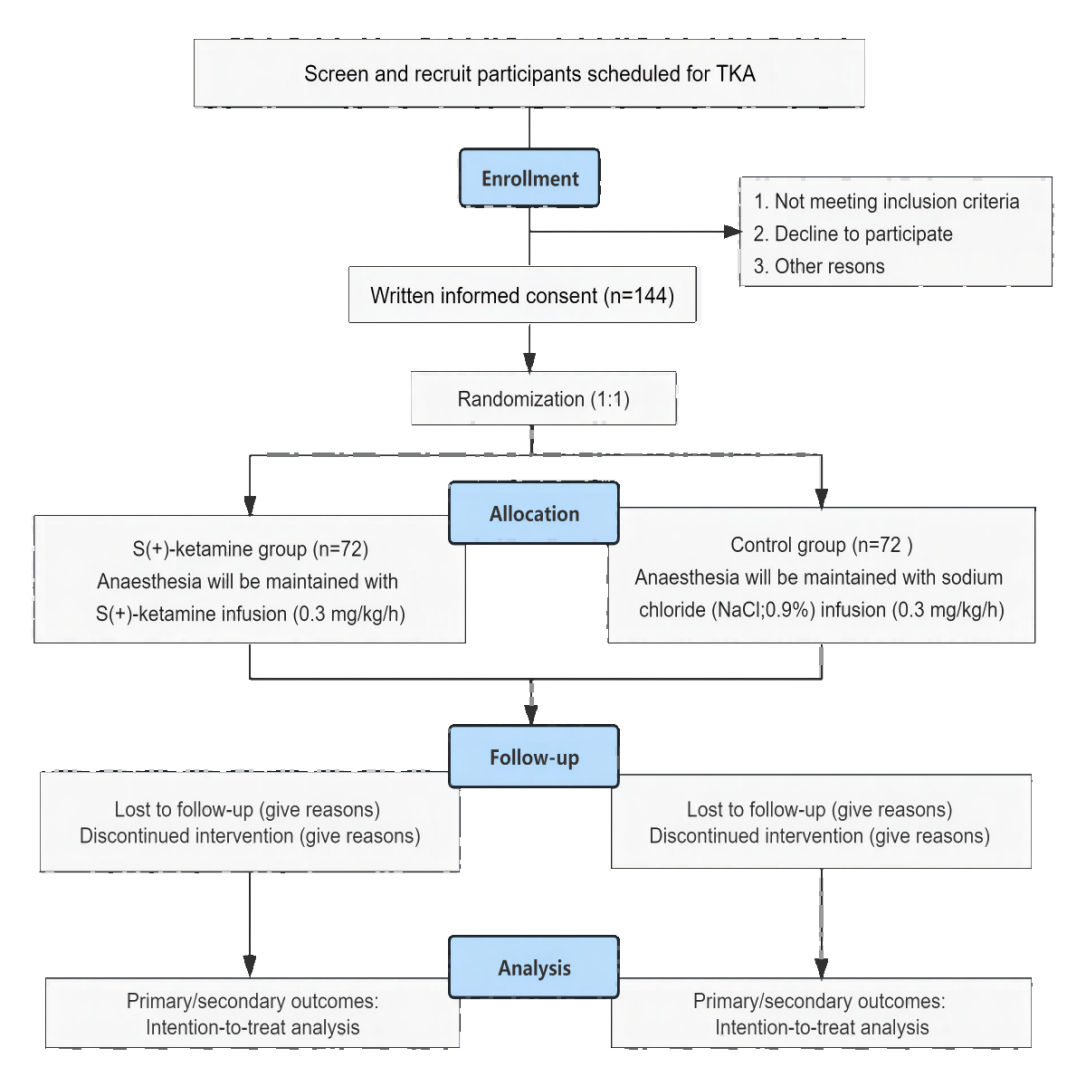
Consolidated standards of reporting trials flow diagram describing patient progress throughout the study. TKA: total knee arthroplasty.

A researcher with a detailed understanding of the study protocol (ie, study objective, inclusion and exclusion criteria, and benefits and risks for patients) will present the protocol to the patients and answer any questions in a concise and comprehensive manner. All patients are required to provide written consent before surgery (Figure 1).

### Inclusion Criteria

The inclusion criteria include patients who have an American Society of Anesthesiologists physical status of I-III; understand the study in detail and voluntarily sign the informed consent form; are to be treated with TKA surgery under general anesthesia; are 65 years or older, regardless of sex; can communicate normally; have no contraindications to drugs, such as midazolam, fentanyl, and S(+)-ketamine; and have a body mass index between 18 kg/m^2^ and 30 kg/m^2^.

### Exclusion Criteria

The exclusion criteria include patients who have increased intracranial or intraocular pressure, severe hypertension, severe psychiatric disease and mental system diseases, severe respiratory diseases, hyperthyroidism, liver or kidney dysfunction, or an allergy to midazolam, fentanyl, or S(+)-ketamine; are unwilling to participate in the study; or misuse alcohol or drugs.

### Intervention

#### Preanesthetic Preparation

An experienced anesthesiologist will participate in the management of the entire perioperative period. Patients will be randomly assigned to the intravenous S(+)-ketamine intervention group or placebo equivalent (0.9% sodium chloride) control group. All patients will be continuously monitored with pulse oximetry, noninvasive blood pressure, and electrocardiogram. General anesthesia induction will be provided after saphenous nerve block is performed with 0.375% ropivacaine (20 ml) by ultrasound guidance.

#### Induction and Maintenance of Anesthesia

All patients will undergo anesthetic induction administered by an experienced anesthesiologist per the protocol using dexamethasone (10 mg), midazolam (0.01-0.05 mg/kg), sufentanil (0.2-0.4 µg/kg), etomidate (0.15-0.3 mg/kg), and atracurium (0.3-0.6 mg/kg). A laryngeal mask airway will be inserted followed by muscle effective relaxation. Patients in both groups will be given ondansetron hydrochloride (8 mg) intravenously during anesthesia induction to prevent postoperative nausea and vomiting.

For patients in the S(+)-ketamine group, anesthesia will be maintained with S(+)-ketamine infusion (0.3 mg/kg/h), sedation will be maintained with propofol infusion (4-12 mg/kg/h), and analgesia will be maintained with remifentanil (0.15-0.3 µg/kg/min). The sufentanil dose at induction and the rate of intraoperative remifentanil and propofol infusions are at the discretion of the anesthesiologist in charge of the patient. The assessment of the depth of anesthesia will be based on clinical evaluation. S(+)-ketamine infusion will be stopped 15 minutes before the end of surgery. Propofol and remifentanil infusion will be stopped at the end of surgery. For the control group, other procedures will be consistent; however, S(+)-ketamine (0.3 mg/kg/h) will be replaced by intravenous sodium chloride (0.9%) infusion (0.3 mg/kg/h).

#### Intraoperative Management

Hypertension will be defined as an intraoperative systolic blood pressure exceeding 20% from the baseline value, and hypotension will be defined as 20% of the baseline value. Norepinephrine should be administered if the patient has hypotension and a heart rate (HR) >90 beats per minute; dopamine should be administered when the patient has hypotension and an HR <50 beats per minute; urapidil hydrochloride should be administered if the patient has hypertension and their HR is normal; and atropine should be administered if the patient has normal blood pressure and a HR <50 beats per minute. The dosages are to be determined by the patient’s sensitivity to drugs to maintain stable circulation.

### Outcome Measures

#### Primary Outcome Measure

The primary outcome is the numeric rating scale (NRS) score at rest and during movement 24 hours after surgery in the surgical ward. The NRS is widely used to assess pain intensity after surgery. The NRS is evaluated on an 11-point scale (no pain: NRS=0; mild pain: 0<NRS<4; moderate pain: 4≤NRS<7; severe pain: 7≤NRS<10; worst pain imaginable: NRS=10).

#### Secondary Outcome Measures

The secondary outcomes are as follows:

1. The NRS score at rest and during movement at 2 hours after surgery.

2. The NRS score at rest and during movement at 48 hours after surgery.

3. The cumulative consumption of opioids, such as pethidine, sufentanil, and morphine, during the first 48 hours after the operation.

4. The number of patients who required additional analgesics, such as opioids (pethidine, sufentanil, or morphine) or nonsteroid anti-inflammatory drugs (acetaminophen or diclofenac) during the first 48 hours after the operation.

5. The total consumption of opioids (pethidine, sufentanil, and morphine) or nonsteroid anti-inflammatory drugs (acetaminophen and diclofenac) during the first 48 hours after the operation.

6. The incidence of nausea and vomiting and adverse central nervous system events at 2, 24, and 48 hours after the operation.

The use of rescue analgesic drugs (types, administration times, and doses) and any adverse side effects (yes or no) will be recorded.

### Participant Timeline

The participant timeline is shown in Table 1.

**Table 1:** Schedule of enrollment, interventions, and assessments for the trial.

			Study period													
			Enrollment		Allocation		Postallocation									Close-out
Time points^a^			–*t_1_*		0		*t_1_*		*t_2_*		*t_3_*		*t_4_*		*t_5_*	
**Enrollment**																
	Eligibility screen	X														
	Informed consent	X														
	Allocation			X												
**Interventions**																
	S(+)-ketamine					X		X		X		X				
	Normal saline					X		X		X		X				
**Assessments**																
	NRS^b^ pain score									X		X		X		
	Postoperative opioid^c^ consumption									X		X		X		
	Patients who required additional analgesics									X		X		X		
	Postoperative nausea									X		X		X		
	Postoperative vomiting									X		X		X		
	Nightmares									X		X		X		
	Hallucinations									X		X		X		
	Dizziness									X		X		X		
	Itchy skin									X		X		X		

^a^The following time points were used: *–t_1_*, preoperative assessment; 0, entering the operating room; *t_1_*, after induction; *t_2_*, end of surgery; *t_3_*, 2 hours after surgery; *t_4_*, 24 hours after surgery; and *T_5_*, 48 hours after surgery.

^b^NRS: numeric rating scale.

^c^Opioids included pethidine, sufentanil, or morphine.

### Safety

The main adverse effects of S(+)-ketamine alone are neuropsychiatric symptoms, such as dizziness. Therefore, S(+)-ketamine is usually used with midazolam and propofol to decrease its psychoactive effects. All adverse events related to this research will be investigated and recorded by research personnel from the time patients sign the informed consent to the end of follow-up. The investigator will decide whether to continue the study according to the status of the patient. Any serious adverse events will be treated with appropriate interventions and submitted to the principal researcher and the Medical Ethics Committee within 24 hours.

### Ethical Considerations

This study has been approved by the Medical Ethics Committee of the First Affiliated Hospital of Shandong First Medical University under approval number YXLL-KY-2021 (077) and will be performed in accordance with the approved guidelines and regulations of the participating institutions. The study was registered at ClinicalTrials.gov (NCT05289050) on February 26, 2022. Informed written consent will be obtained from all patients before inclusion in the study. Because the involvement of the patients is voluntary, the study can be stopped at any time.

### Data Management

The basic information of the patients before surgery and the dosage of drugs used during the operation will be collected by the anesthesiologists participating in surgical anesthesia, and the follow-up data after surgery will be provided by independent researchers. Each data point related to the study will be entered into the case report form to improve data completeness and accuracy, and electronic data will be collected and analyzed by statisticians. All the clinical trial materials will be stored securely.

### Statistics

The SD will be described for measurement data that follow a normal distribution, and the median (IQR) will be described for measurement data that do not conform to a normal distribution. The student *t* test will be used to compare continuous and normally distributed variables, and the Mann-Whitney U test will be used to compare nonnormally distributed variables. Counts (percentages) will be described for categorical variables using χ^2^ analysis or Fisher exact tests for compilation.

Both primary and secondary outcomes will be based on an intention to treat analysis. SPSS 22.0 software (IBM) will be used for statistical analysis, and a *P* value of <.05 will be considered to be statistically significant.

### Sample Size

The minimum clinically important difference (MCID) can be used to guide the noninferiority margin for clinical trials and to determine the sample size in a clinical trial [16,17]. The NRS pain score ranges from 0 (no pain) to 10 (worst pain imaginable). The MCID for the NRS that was used for the calculation of the study sample size was 1.3; however, some studies suggest that an MCID of 1.5 or 2 is appropriate [18-20]. The sample size would have been too small if we choose an MCID of 2. Considering a 10% withdrawal or dropout rate, a sample size of 72 patients per group was calculated, with a power of 80%. The MCID determined 1.5 as statistically significant, with an SD of 3 at the 5% bilateral significance level.

## Results

The protocol was registered at ClinicalTrials.gov (NCT05289050) on February 26, 2022. It was performed in accordance with the approved guidelines and regulations of the participating institutions. Recruitment began in April 2022. Data collection is expected to conclude in September 2024. Study completion is expected in December 2024. As of December 2023, we have enrolled 132 patients.

## Discussion

TKA is increasingly performed in geriatric patients to cure pain, deformation, and the inability to walk normally caused by knee arthritis. The total number of TKA procedures may increase by 401% in 2040 compared to that in 2014 [21]. Poor management of acute pain after TKA increases the risk of complications, such as venous thrombosis, an inability to exercise effectively, and pneumonia. Currently, there is a consensus that inadequate postoperative pain control measures are being implemented, especially in older patients. Opioids are the main treatment for relieving TKA postoperative pain [22]. Considering the side effects of opioids, appropriate multimodal medication projects are required to decrease postoperative opioid use and improve effects with decreasing pain levels in geriatric patients. Ketamine can suppress the pathway of central sensitization and secondary postoperative hyperalgesia and has been proposed as a component of multimodal analgesia for various surgical procedures [23]. Ketamine in subanaesthetic doses is available as an adjuvant to the standard regimen of opioids, presenting prominent analgesic and opioid-sparing effects without adverse effects [24]. Some studies have shown that low-doses of S(+)-ketamine can reduce the incidence of side effects (eg, the induction of hypotension) and high doses of opioids through its sympathetic stimulation, analgesia, and antagonism of the NMDA receptor [25,26]. Similarly, a meta-analysis demonstrated that the administration of low-dose ketamine can decrease total opioid use and pain scores over 2 days and may also reduce postoperative nausea and vomiting. This has been attributed to reductions in total opioid administration, leading to decreased opioid-associated nausea and vomiting [27,28]. Another meta-analysis demonstrated that perioperative intravenous ketamine reduced postoperative opioid consumption by 8 mg morphine equivalents per day [29].

Additional studies have demonstrated that a low-dose intravenous infusion of ketamine can be administered as an adjuvant drug to alleviate acute postoperative pain, while the guidelines for ketamine for postoperative acute pain have found grade B evidence to advocate perioperative ketamine as an adjuvant [15,30].

Although low-dose S(+)-ketamine has been shown to be beneficial in pain management after various surgeries, there is no consensus regarding the effectiveness of an S(+)-ketamine analgesic combined with opioids in older patients with knee arthroplasty. In addition, the appropriate administration duration and dose of S(+)-ketamine to achieve pain relief in older patients are unknown. A randomized controlled trial will be conducted to assess the efficacy and safety of an intraoperative intravenous low-dose of S(+)-ketamine for pain management in older patients undergoing TKA.

The design of our protocol has several limitations. First, this is a single-center study with a relatively small sample size, and only older patients undergoing TKA will be enrolled, which limits the generalizability of our outcomes. Second, the end of our follow-up is 48 hours postoperatively. A longer follow-up is necessary to demonstrate the long-term effects and safety of S(+)-ketamine.

In conclusion, we will appraise the efficacy and safety of the intraoperative intravenous injection of S(+)-ketamine to relieve acute pain after TKA in older patients who may benefit from the administration of S(+)-ketamine.

## Abbreviations

HR: heart rate
MCID: minimum clinically important difference
NMDA: N-methyl-D-aspartate receptor
NRS: numeric rating scale score
TKA: total knee arthroplasty

## Data Availability

The data sets used or analyzed during the trial will be made available from the corresponding author on reasonable request.
